# Associations of Complete Blood Count Parameters with Disease-Free Survival in Right- and Left-Sided Colorectal Cancer Patients

**DOI:** 10.3390/jpm12050816

**Published:** 2022-05-18

**Authors:** Alhasan Alsalman, Mohammad A. Al-Mterin, Ala Abu-Dayeh, Ferial Alloush, Khaled Murshed, Eyad Elkord

**Affiliations:** 1 Natural and Medical Sciences Research Center, University of Nizwa, Nizwa 616, Oman; hasan.alsalman@unizwa.edu.om (A.A.); mohammed.mterin@unizwa.edu.om (M.A.A.-M.); 2 Department of Pathology, Hamad Medical Corporation, Doha P.O. Box 3050, Qatar; aabudayeh@hamad.qa (A.A.-D.); falloush@hamad.qa (F.A.); kmurshed@hamad.qa (K.M.); 3 Biomedical Research Center, School of Science, Engineering and Environment, University of Salford, Manchester M5 4WT, UK

**Keywords:** CRC, biomarkers, complete blood count, DFS

## Abstract

Colorectal cancer (CRC) is a leading cause of cancer-related deaths worldwide. Some complete blood count (CBC) parameters are found to be associated with CRC prognosis. In this study, ninety-seven pretreated CRC patients were included, and the patients were divided into two groups: left-sided and right-sided, depending on the anatomical location of the tumor. Based on clinicopathologic features including tumor budding, disease stages, and tumor anatomical location, levels of CBC parameters were compared, and disease-free survivals (DFS) were determined. There were differences between patients with different tumor budding scores for only three parameters, including red cell distribution width (RDW), numbers of platelets, and mean platelet volume (MPV). Furthermore, numbers of WBCs, monocytes, and MPV in CRC patients with early disease stages were higher than those with advanced stages. However, levels of eosinophil in CRC patients with advanced stages were higher than those with early stages. Depending on the tumor anatomical location, we observed that numbers of red blood cells (RBCs), hemoglobin (Hgb), and hematocrit (Hct) in CRC patients with left-sided tumors were higher than those with right-sided tumors. We found that low levels of MPV were associated with shorter DFS. However, high levels of eosinophils were associated with shorter DFS in all CRC patients. When patients were divided based on the tumor anatomical location, higher levels of MPV, MCHC, and Hgb were associated with better DFS in the left-sided but not right-sided CRC patients. However, left-sided, but not right-sided, CRC patients with high levels of eosinophil and RDW had shorter DFS. Furthermore, right-sided, but not left-sided, CRC patients with high levels of platelets tended to have a shorter DFS. Our data show that MPV and eosinophils could serve as potential prognostic biomarkers in pre-treatment CRC patients, regardless of the tumor anatomical location. Additionally, lower levels of MPV, MCHC, and Hgb, and high levels of eosinophils and RDW could be negative predictive biomarkers in left-sided CRC patients.

## 1. Introduction

Colorectal cancer (CRC) is a leading cause of cancer-related deaths worldwide, representing 8% of cancer mortality [1,2]. Development and progression of CRC are associated with a number of factors, including age, diet, hereditary polyposis syndrome, and inflammatory bowel disease [3,4]. Cancer-associated inflammation has been related to disease development and prognosis in many cancers, including CRC [5]. Therefore, routinely inflammatory blood biomarkers could be used as prognostic factors for overall and disease-free survival in cancer patients [5,6]. Variations in the epidemiology of right-sided and left-sided CRC might originate from the existence of various pro-carcinogenic factors in the ascending versus descending colon [7]. TNM classification does not accurately represent the prognosis of colorectal cancer patients (CRC), and there are significant differences between patients with similar TNM stages [8,9]. However, TNM stage classification and tumor site have long-guided the use of surgery, chemotherapy, and/or radiation treatment for CRC patients [8].

Survival rates of CRC patients are influenced by the stage of the disease and the presence of metastases. While early-stage patients with localized cancer have a 90% 5-year survival rate, intermediate (regional invasive tumors) patients have a 70% 5-year survival rate, and advanced-stage patients with distant metastases have a 10% 5-year survival rate [4]. Considering the stage as a reference standard for CRC, however, methods to increase the predictive value of CRC patients’ survival are still needed [10]. Therefore, it is critical to find prognostic biomarkers for CRC patients to improve patients’ survival. 

Determination of ideal biomarkers for CRC remains problematic, despite several efforts to develop reliable biomarkers [11,12]. Unfortunately, many investigated biomarkers have restrictions, hindering their use in clinical practice, such as limited diagnostic and prognostic accuracy, long turnaround times, and high costs [13].

Complete blood count is one of the critical laboratory tests [14]. It provides a wealth of information about the blood cells and the immune system by evaluating different parameters. Moreover, it has different advantages, such as being cheap, simple to perform, and availability in different departments, from the emergency room to the critical care unit. Therefore, it is critical to utilize this laboratory test to provide better care for patients [14]. This study aimed to define predictive biomarkers for CRC patients, which are low-cost and easily obtainable through routine blood counts.

## 2. Materials and Methods

### 2.1. Sample Collection and CBC Analyses

This study was performed under ethical approval (protocol No. MRC-02-18-012) from the Medical Research Center, Hamad Medical Corporation, Doha, Qatar. Research was conducted in compliance with the applicable standards and regulations. Patients involved in this study did not receive any anti-cancer treatments prior to surgery, and they provided written informed consent prior to sample collection. The clinical and pathological characteristic features of patients are described in Table 1. Whole blood samples for complete blood count (CBC) were collected from 97 CRC patients scheduled for operation one to two weeks prior to surgery. Samples were collected in the morning between 7:00 and 9:00 am. Regarding the batching, all CBC parameters were analyzed in the same run for each sample. However, not all samples were run in the same batch, as the samples were collected on different days of the week for approximately two years. The samples were sent immediately to the Rapid Response Lab at Hamad Medical Corporation (Doha, Qatar) for CBC analyses within two hours of collection. Therefore, all samples were exposed to the same conditions and were all analyzed at the same time of the day (morning time) using the same analyzers (XP-300 Automated Hematology Analyzer, Sysmex Corporation (Kobe, Hyogo, Japan)). The analyzers were validated and verified based on strict standards, including precision, accuracy, reference range, linearity, interfering substance, limits of detection, and carryover. All the quantified data were in the linearity range. Reference ranges for the linearity and detection limits for the hematology analyzer used for CBC parameters are shown in Supplementary Table S1.

Comprehensive internal and external quality controls are always performed in labs at Hamad Medical Corporation. Within-run precision (reproducibility) was performed using a single run of 20 measurements and is reported as a coefficient of variation. Between-batch precision was performed on a single measurement that was repeated every day for a 20-day period. Stabilized quality control samples were used for between-batch precision. In addition to that, the participation in an External Quality Assessment (EQA) Scheme (proficiency testing) was mandatory. The EQA samples were handled as routine patient samples, and samples that resemble pathological samples were used. The EQA results were used to compare the results with international consensus and reference results.

In January 2022, disease-free survival (DFS) data for the 97 patients were collected. Disease progression occurred in 16 out of the 97 patients in the form of tumor local recurrence or development of new lymph node and/or distant metastasis. Disease progression was assessed by a contrast-enhanced chest-abdomen-pelvis computed tomography (CT) scan that was performed for patients on their clinical follow-up.

### 2.2. Statistical Analyses

GraphPad Prism 9 software (GraphPad Software, San Diego, CA, USA) was used to perform statistical analyses. A one-way ANOVA test and nonparametric counterpart of ANOVA (Kruskal-Wallis test) were used to determine statistical significance in grouped analyses. On samples that passed the Shapiro-Wilk normality test, unpaired t-tests were used to compare groups of normally distributed data, while Mann-Whitney tests were used for samples that did not show normal distribution.

The Shapiro-Wilk test was used to determine the normality of datasets. All CBC parameters were divided into low and high groups based on below/above mean for normally distributed data and below/above median for non-normally distributed data. Mean or median were used as cut-off values for all CBC parameters to categorize patients into high and low groups. The Kaplan-Meier method was used to predict DFS, and the log-rank test was utilized to analyze differences in DFS between groups.

## 3. Results

### 3.1. Differential Levels of Red Cell Distribution Width (RDW), Platelet, and Mean Platelet Volume (MPV) in CRC Patients with Varying Tumor Budding Status

**Figure 1:**
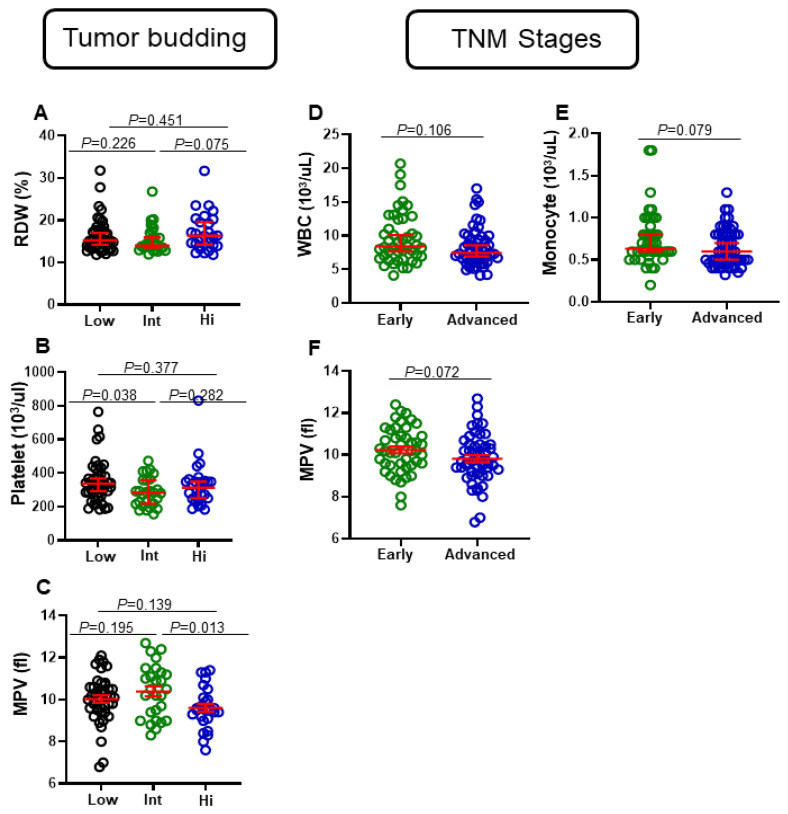
Comparison of some CBC parameters based on tumor budding and TNM stages of CRC patients. Patients were divided into three groups based on degree of tumor budding: low, intermediate, and high. Scatter plots show differences in levels of RDW (**A**), platelets (**B**), and MPV (**C**). Additionally, patients were divided into two groups based on TNM stages (early and advanced). Scatter plots show differences in levels of WBCs (**D**), monocytes (**E**), and MPV (**F**). Red lines represent median ± 95% CI or mean ± SEM.

Complete blood count (CBC) is one of the most common laboratory tests performed today. These tests are essential to diagnose different diseases, such as anemia, certain cancers, infections, immunodeficiency, and allergies [14]. Tumor budding is associated with bad prognosis in colorectal cancer patients [15]. Based on the tumor budding score, we divided our cohort into three groups: low, intermediate, and high. Then, we compared levels of various clinical parameters between the three groups. We did not observe any significant differences between levels of white blood cells (WBC), RBC, Hgb, Hct, mean corpuscular volume (MCV), mean corpuscular hemoglobin (MCH), mean corpuscular hemoglobin concentration (MCHC), neutrophils, lymphocytes, monocytes, eosinophils, and basophils between patients with different tumor budding statuses. Among all CBC parameters, only three were observed to have differential levels between patients with different tumor budding statuses (Figure 1A–C). We found that levels of RDW in CRC patients with high tumor budding were higher than CRC patients with intermediate status, (median (95% CI), 16.3 (15.4–19.00) vs. 14 (14.1–16.6), *p* = 0.075), but not with low status (Figure 1A). Moreover, numbers of platelets in CRC patients with low tumor budding status were significantly higher than CRC patients with intermediate status (median (95% CI), 336 (309.6–388.7) vs. 284 (252.1–320.9), *p* = 0.038), but not with high status (Figure 1B). This result could be skewed due to four CRC patients with higher platelet values. Additionally, numbers of MPV in CRC patients with intermediate tumor budding status were significantly higher than CRC patients with high status (mean ± SEM, 10.39 ± 0.24 vs. 9.61 ± 0.19, *p* = 0.013), but not with low tumor budding status (Figure 1C).

### 3.2. Differential Levels of Eosinophils, WBC, Monocytes, and MPV in CRC Patients with Different Tumor Stages

We then compared the levels of these clinical parameters between CRC patients with early and advanced pathologic stages. We did not observe any significant differences in levels of RBC, Hgb, Hct, MCV, MCH, MCHC, neutrophils, lymphocytes, monocytes, basophils, and eosinophils between patients with different tumor stages. Interestingly, WBC numbers in patients with early stages were higher, compared to those with advanced tumor stages (median (95% CI), 8.40 (8.49–10.78) vs. 7.45 (7.52–9.14), *p* = 0.106) (Figure 1D). Moreover, monocyte numbers in CRC patients with early tumor stages were higher, compared to those with advanced tumor stages (median (95% CI), 0.63 (0.649–0.841) vs. 0.6 (0.582–0.708), *p* = 0.07) (Figure 1E). Additionally, in patients with early stages, numbers of MPV were higher, compared to patients with advanced stages (median (95% CI), 10.2 (9.90–10.56) vs. 9.7 (9.48–10.15), *p* = 0.072) (Figure 1F).

### 3.3. Differential Levels of RBC, Hgb, Hct, RDW, and Lymphocytes in Right-Sided and Left-Sided CRC Patients

**Figure 2:**
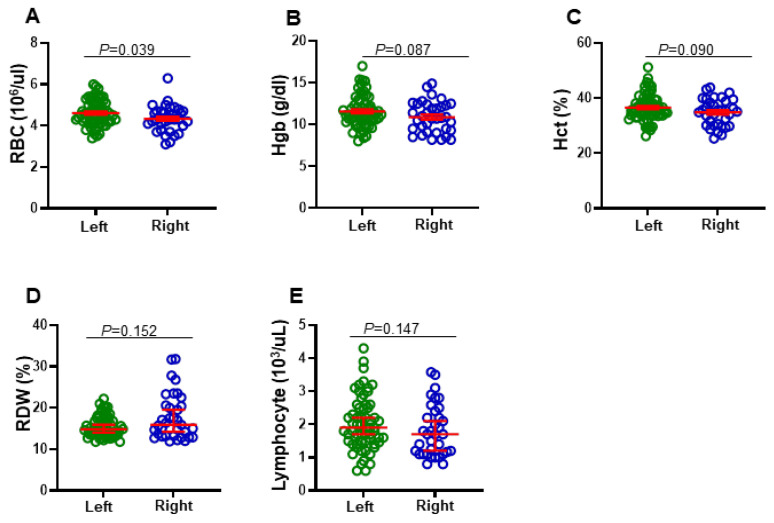
Comparison of some CBC parameters based on the tumor anatomical location of CRC patients. Patients were divided into two groups based on left-sided and right-sided tumors. Scatter plots show the differences in levels of RBC (**A**), Hgb (**B**), Hct (**C**), RDW (**D**), and lymphocytes (**E**). Red lines represent median ± 95% CI or mean ± SEM.

We then went further and compared between levels of clinical parameters according to tumor location (right-sided and left-sided). Among all CBC parameters, we found that levels of RBC, Hgb, and Hct in left-sided CRC patients were higher than in right-sided CRC patients (mean ± SEM, 4.61 ± 0.07 vs. 4.34 ± 0.10, *p* = 0.039; 11.58 ± 0.24 vs. 10.90 ± 0.31, *p* = 0.087; 36.57 ± 0.617 vs. 34.81 ± 0.817, *p* = 0.090, respectively) (Figure 2A–C). Additionally, we found that levels of RDW in right-sided CRC patients were higher than in left-sided CRC patients (median (95% CI), 15.90 (15.89–19.71) vs. 14.80 (14.84–16.17), *p* = 0.152) (Figure 2D). Furthermore, lymphocyte numbers in left-sided CRC patients were higher than in right-sided CRC patients (median (95% CI), 1.90 (1.82–2.23) vs. 1.70 (1.54–2.08), *p* = 0.147) (Figure 2E).

### 3.4. Clinicopathological Parameters and Disease-Free Survival

**Figure 3:**
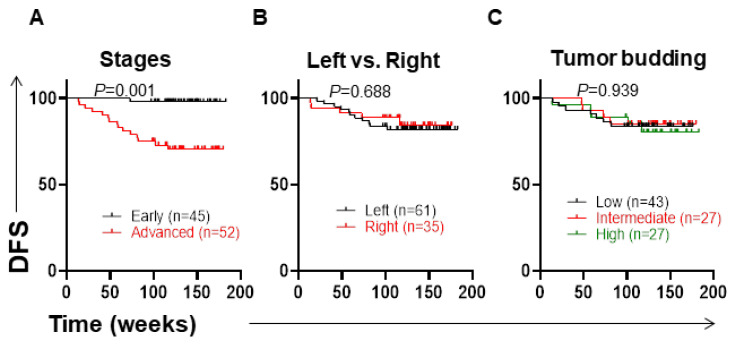
Kaplan–Meier curves of disease-free survival (DFS) based on TNM stages, anatomical location, and tumor budding. Patients were divided into groups based on stages (early and advanced) (**A**), anatomical location (left and right) (**B**), and tumor budding (low, intermediate, high) (**C**), and DFS was determined for these groups.

The characteristic features of CRC patients are shown in Table 1. The TNM staging system is critical to determine the treatment of CRC patients [8]. As expected in this cohort, we found that CRC patients with advanced stages (III and IV) had significantly shorter DFS, compared to patients with early stages (I and II) (*p* = 0.0005) (Figure 3A).

The pathogenesis of CRC is based on the tumor’s anatomical location and varies between the left and right sides of the colon, depending on the molecular and histological features [4]. All patients (n = 96) were divided into two groups: left side (n = 61) and right side (n = 35) of the colon. We did not find any difference in DFS between left-sided and right-sided CRC patients (*p* = 0.688) (Figure 3B).

Tumor budding is an important prognostic factor for patients with CRC. Based on different studies, tumor budding has been correlated with poor clinical outcomes in CRC patients [15,16]. In our study, we did not find any difference in DFS between low, intermediate, and high tumor budding in CRC patients (*p* = 0.939) (Figure 3C).

### 3.5. Low Levels of MPV and High Numbers of Eosinophils Are Associated with Shorter DFS

**Figure 4:**
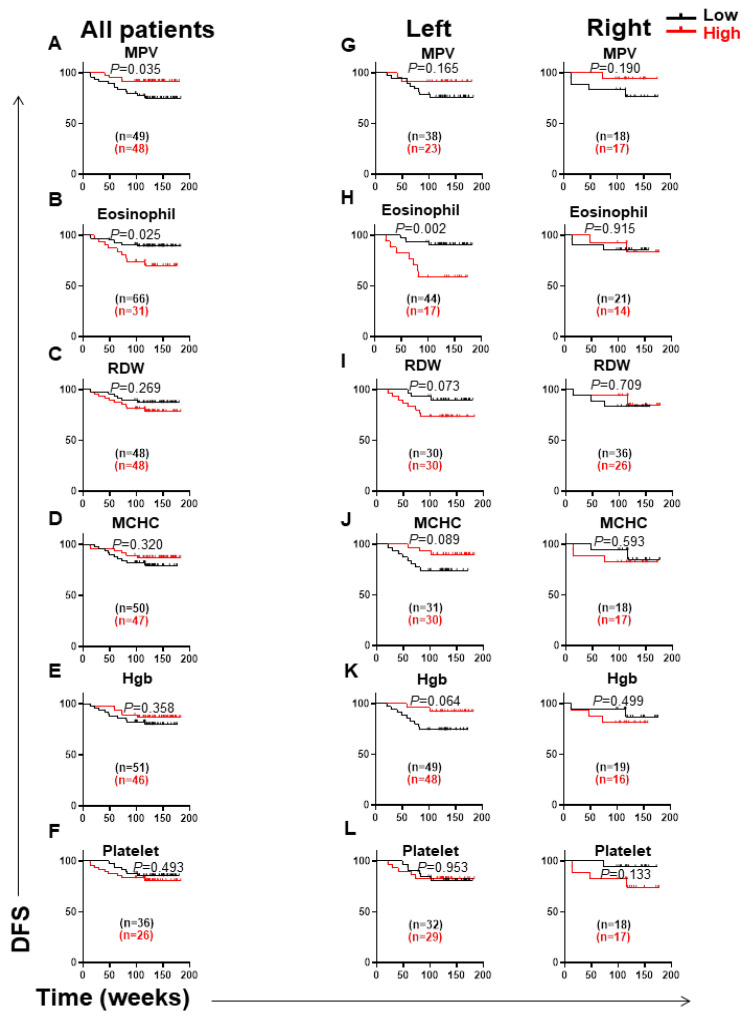
Kaplan–Meier curves of disease-free survival (DFS) based on different CBC parameters, including MPV, eosinophils, RDW, MCHC, Hgb, and platelet. Patients were divided into high and low groups for MPV (**A**), eosinophils (**B**), RDW (**C**), MCHC (**D**), Hgb (**E**), and platelet (**F**), and DFS was determined for these groups. Additionally, sub-analyses for left-sided and right-sided groups were performed for these six parameters, as shown in (**G**–**L**).

We then investigated the association between CBC parameters and DFS for all CRC patients. Among all CBC parameters, only MPV and eosinophils showed significant associations with DFS for all patients (Figure 4A,B). MPV is the most commonly used parameter to measure the average size of platelets, and it is an alternative indicator of platelet activation [17]. In our study, the cut-off value for MPV was 10.01. Patients were divided into two groups: one group with MPV less than 10.01 fL (low) and another group with MPV more than 10.01 (high). We found that low MPV levels were significantly associated with shorter DFS for all CRC patients (*p* = 0.035) (Figure 4A). Eosinophils are thought to be multifunctional mobile cells that control and regulate various biological processes and responses in health and disease [18]. The cut-off value for eosinophils was determined using the median, which was 0.2%. We found that patients with high levels of eosinophils had significantly shorter DFS than patients with lower levels of eosinophils (*p* = 0.025) (Figure 4B). Additionally, there were no significant associations between levels of RDW, MCHC, Hgb, and platelet with DFS in all CRC patients (Figure 4C–F).

### 3.6. Associations of CBC Parameters with DFS in Left-Sided and Right-Sided CRC Patients

All CRC patients were divided into left-sided and right-sided groups, and we performed sub-analyses for these two groups by investigating the associations between CBC parameters and DFS in each group, separately. Cut-off values for CBC parameters were determined based on the mean for normally distributed data or median for non-normally distributed data. For the left-sided CRC patients, the cut-off values for MPV, RDW, MCHC, Hgb, and platelets were 10.4, 14.8, 31.3, 11.58, and 295, respectively, while for the right-sided CRC patients, these cut-off values were 9.9, 15.9, 31.5, 10.9, and 313, respectively. Patients with lower levels of MPV showed a trend towards shorter DFS, compared to patients with higher levels of MPV in both left-sided (*p* = 0.165) and right-sided (*p* = 0.190) CRC patients (Figure 4G). Interestingly, we found that high numbers of eosinophils in the left-sided, but not in the right-sided, CRC patients were associated with shorter DFS (*p* = 0.002) (Figure 4H). Additionally, left-sided, but not right-sided, CRC patients with high levels of RDW tended to have shorter DFS (*p* = 0.073) (Figure 4I). Moreover, lower levels of MCHC (*p* = 0.089) and Hgb (*p* = 0.064) showed trends towards shorter DFS in the left-sided but not in the right-sided CRC patients (Figure 4J,K). On the other hand, higher platelet numbers in the right-sided but not in the left-sided CRC patients showed a trend towards shorter DFS (*p* = 0.133) (Figure 4L).

## 4. Discussion

Biomarkers play vital roles in the identification and management of cancer patients. In CRC, there is an increasing interest in biomarker development and validation according to specific tumor types [19]. Moreover, accurate and robust prognostic biomarkers could identify the high-risk CRC patients at early stages, improving their cure chances [20]. Disease stage and anatomical status are important factors in determining disease progression and overall survival (OS) of CRC patients [4,21,22]. Some studies reported that left-sided CRC patients with early stages (I and II) had significantly poorer survival, compared with right-sided colon cancer patients [4]. However, the prognosis of advanced stages (III and IV) in the right-sided group of colon cancer patients was worse compared to left-sided [23,24]. 

Altered levels of MPV could be helpful as a prognostic biomarker for malignant tumor patients; however, the association between MPV levels and patient outcome remains unclear [25]. Compared with healthy controls, MPV levels were elevated in CRC patients, and their levels were decreased following surgery [26]. Therefore, MPV could be a simple way to monitor post-operative CRC patients. Many studies reported the association of MPV levels with different types of cancers, where reduced MPV levels were significantly associated with poor outcomes in esophageal, breast, lung, renal, and bladder cancers [27,28,29,30,31]. However, a recent study reported that lower levels of MPV were correlated with higher tumor stages, and with worse prognosis in CRC patients [32]. Additionally, Chang et al. reported that low levels of MPV were associated with shorter PFS in the metastatic CRC patients undergoing first-line chemotherapy [25]. In agreement with these studies, we found that lower MPV levels were associated with shorter DFS for all CRC patients. We observed that both left-sided and right-sided CRC patients with low MPV levels tended to have shorter DFS. In addition, we found that levels of MPV in CRC patients with early stages were higher than in patients with advanced stages.

The role of eosinophils in the development and progression of cancer is unclear. Eosinophils could play different roles in different types of cancers. Some studies reported that lower numbers of tumor-infiltrating eosinophils in CRC patients were associated with an increased disease risk and worse prognosis [10,33,34]. In the circulation of CRC patients, low levels of eosinophils were associated with reduced incidence of CRC development and better prognosis [35,36]. However, Xiong et al. found that tumors with a low number of M1 macrophages or a high number of M2 macrophages, eosinophils, and neutrophils were associated with a poor prognosis [37]. Additionally, eosinophilia has been associated with a worse prognosis in different types of cancers, such as breast cancer, hematological malignancies, and myelodysplastic syndromes (MDSs) [38,39,40]. Overall, evidence suggests that low levels of eosinophils are related to a good prognosis in tumor tissues [36]. In contrast, eosinophilia in peripheral blood is associated with a poor prognosis in cancer patients [36,41]. We found that high levels of eosinophils were associated with worse DFS in all CRC patients. An interesting novel finding in this study was that high levels of eosinophils in the left-sided, but not in the right-sided, CRC patients were significantly associated with worse DFS.

Red blood cell distribution width (RDW) is a simple and low-cost marker that could be used for clinical purposes. It is a traditional biomarker for RBC size variation and an indication of erythrocyte homeostasis [42]. In most tumor types and stages, high pretreatment RDW levels were associated with poor clinical outcomes [43]. Studies reported that increased levels of RDW were associated with poor prognosis in different types of cancers, such as esophageal cancer [44,45,46], lung cancer [47,48], and hematological malignancies [49,50]. In CRC patients, higher levels of RDW were significantly associated with worse prognosis, particularly in metastatic CRC patients [51]. This significant correlation in CRC could be attributed to persistent inflammation and cancer-related anemia [43]. Ay et al. found that levels of RDW were significantly increased in CRC patients compared with colon polyp patients [52]. A recent study reported that higher levels of RDW were associated with worse DFS and OS in the early stages (I and II) of CRC patients after curative resection [53]. Therefore, RDW has the potential to be utilized as an early-warning biomarker for colon cancers. Interestingly, we observed that higher levels of RDW were associated with worse DFS only in the left-sided, but not in the right-sided, CRC patients. 

Hgb and MCHC levels were significantly lower in peripheral blood of CRC patients [54]. Moreover, lower levels of Hgb and MCHC could be associated with worse DFS and OS [54]. Additionally, Tampellini et al. reported that higher Hgb levels were associated with a higher response rate, longer time to progression, and higher survival rate than anemic patients [55]. In our study, we observed that levels of Hgb in patients with left-sided tumors were higher than in patients with right-sided tumors. Furthermore, we found that lower levels of Hgb and MCHC were associated with shorter DFS in the left-sided but not in the right-sided CRC patients.

Platelets are involved in a variety of cancer development and metastasis pathways [32]. Previous studies reported that higher levels of platelets were associated with metastasis, carcinogenesis, and angiogenesis in solid cancers [56,57]. Moreover, increased platelets were associated with worse OS and prognosis in different types of cancers, including pancreatic, gastric, colorectal, endometria, and ovarian cancers [58,59,60,61]. Interestingly, we observed that right-sided, but not left-sided, CRC patients with high platelet levels tended to have shorter DFS. To date, this is the first study that investigated the association between CBC parameters and DFS in the left-sided and right-sided CRC groups independently. Although this study highlights important findings regarding the role of CBC parameters in predicting progression of CRC patients, there are some limitations. It is a study with a relatively small sample size, including 97 patients. Therefore, multi-center investigations are required to confirm these findings in larger cohorts of patients. Moreover, future studies are needed to investigate the underlying mechanisms of Hgb, MCHC, and RDW in the progression of left-sided CRC patients, in addition to eosinophils and MPV in the progression of all CRC patients. 

## 5. Conclusions

Our data suggest that CBC parameters play different roles in DFS of CRC patients. Interestingly, we found that the lower levels of MPV, MCHC, and Hgb and higher levels of eosinophils and RDW were associated with shorter DFS in the left-sided CRC patients. Additionally, higher platelet levels were associated with worse DFS in the right-sided CRC patients. In this study, we showed that some CBC parameters could be useful for predicting DFS in pre-treatment CRC patients, and could be widely utilized in daily clinical practice as routinely available, simple, effective, less expensive, and repeatable tests. However, their significance should be examined in larger cohorts and longer follow-up times. Additionally, it would be interesting to follow-up this cohort of patients and evaluate the associations between CBC parameters and OS. Moreover, we plan to investigate the association between different biochemical tests with OS and DFS in this cohort of CRC patients. 

## Figures and Tables

**Table 1:** Characteristic features of colorectal cancer patients.

	CRC Patients
**Number**	97
**Median age (range)**	59 (18–96)
**Gender** (Male:Female)	65:32
**TNM stage**	
I	10 (0) §
II	35 (1) §
III	39 (8) §
IV	13 (7) §
**Tumor budding**	
Low	43 (7) §
Intermediate	27 (4) §
High	27 (5) §
**Right/Left-sided**	61 (11) §
Left	
Right	35 (5) §

CRC, Colorectal cancer. §: Number of patients who had disease progression after treatment.

## Data Availability

The data presented in this study are available on request from the corresponding author.

## Supplementary Materials

The following supporting information can be downloaded at: https://www.mdpi.com/article/10.3390/jpm12050816/s1. Table S1: Reference ranges for the linearity and detection limits for the hematology analyzer used for CBC parameters.

## Abbreviations

CBC	complete blood count
CRC	colorectal cancer
DFS	disease-free survival
Hgb	hemoglobin
Hct	hematocrit
MPV	mean platelet volume
MCH	mean corpuscular hemoglobin
MDSs	myelodysplastic syndromes
OS	overall survival
RDW	red cell distribution width
RBC	red blood cells
TNM	tumor, nodes, and metastases
WBC	white blood cells.
