# Supplementary material for: Associations of Complete Blood Count Parameters with Disease-Free Survival in Right- and Left-Sided Colorectal Cancer Patients
Source: J Pers Med. 2022 May 18;12(5):816. doi: 10.3390/jpm12050816 (PMC9146340; doi:10.3390/jpm12050816)
Supplement: Supplementary file 1 [file jpm-12-00816-s001.zip › jpm-1698437-supplementary.pdf]

Supplementary Table S1: Reference ranges for the linearity and detection limits for the hematology analyzer used for CBC parameters.

Parameter	Units	Linearity	Detection limit
WBC	x10^3 cells/µL	1.0 - 99.9	1.0
RBC	x10^6 cells/µL	0.3 - 7.00	0.3
HGB	g/dl	0.1 - 25	0.1
HCT	%	10 - 60	10
PLT	x10^3 cells/µL	10 - 999	10
